# Radiomics based on fluoro-deoxyglucose positron emission tomography predicts liver fibrosis in biopsy-proven MAFLD: a pilot study

**DOI:** 10.7150/ijms.64458

**Published:** 2021-09-07

**Authors:** Zhong-Wei Chen, Kun Tang, You-Fan Zhao, Yang-Zong Chen, Liang-Jie Tang, Gang Li, Ou-Yang Huang, Xiao-Dong Wang, Giovanni Targher, Christopher D. Byrne, Xiang-Wu Zheng, Ming-Hua Zheng

**Affiliations:** 1 Department of Radiology, the First Affiliated Hospital of Wenzhou Medical University, Wenzhou, China.; 2 Department of Nuclear Medicine, the First Affiliated Hospital of Wenzhou Medical University, Wenzhou, China.; 3 NAFLD Research Center, Department of Hepatology, the First Affiliated Hospital of Wenzhou Medical University, Wenzhou, China.; 4 Key Laboratory of Diagnosis and Treatment for the Development of Chronic Liver Disease in Zhejiang Province, Wenzhou, China.; 5 Section of Endocrinology, Diabetes and Metabolism, Department of Medicine, University and Azienda Ospedaliera Universitaria Integrata of Verona, Verona, Italy.; 6 Southampton National Institute for Health Research Biomedical Research Centre, University Hospital Southampton, Southampton General Hospital, Southampton, UK.; 7 Institute of Hepatology, Wenzhou Medical University, Wenzhou, China.

**Keywords:** Metabolic dysfunction-associated fatty liver disease, Fibrosis, Radiomics, ^18^F-FDG PET/CT

## Abstract

**Rationale:** Since non-invasive tests for prediction of liver fibrosis have a poor diagnostic performance for detecting low levels of fibrosis, it is important to explore the diagnostic capabilities of other non-invasive tests to diagnose low levels of fibrosis. We aimed to evaluate the performance of radiomics based on ^18^F-fluorodeoxyglucose (^18^F-FDG) positron emission tomography (PET) in predicting any liver fibrosis in individuals with biopsy-proven metabolic dysfunction-associated fatty liver disease (MAFLD).

**Methods:** A total of 22 adults with biopsy-confirmed MAFLD, who underwent ^18^F-FDG PET/CT, were enrolled in this study. Sixty radiomics features were extracted from whole liver region of interest in ^18^F-FDG PET images. Subsequently, the minimum redundancy maximum relevance (mRMR) method was performed and a subset of two features mostly related to the output classes and low redundancy between them were selected according to an event per variable of 5. Logistic regression, Support Vector Machine, Naive Bayes, 5-Nearest Neighbor and linear discriminant analysis models were built based on selected features. The predictive performances were assessed by the receiver operator characteristic (ROC) curve analysis.

**Results:** The mean (SD) age of the subjects was 38.5 (10.4) years and 17 subjects were men. 12 subjects had histological evidence of any liver fibrosis. The coarseness of neighborhood grey-level difference matrix (NGLDM) and long-run emphasis (LRE) of grey-level run length matrix (GLRLM) were selected to predict fibrosis. The logistic regression model performed best with an AUROC of 0.817 [95% confidence intervals, 0.595-0.947] for prediction of liver fibrosis.

**Conclusion:** These preliminary data suggest that ^18^F-FDG PET radiomics may have clinical utility in assessing early liver fibrosis in MAFLD.

## Introduction

Metabolic dysfunction-associated fatty liver disease (MAFLD), formerly named non-alcoholic fatty liver disease (NAFLD), is becoming the most common chronic liver disease and threatening people's health seriously 1-3. Disease severity of MAFLD should be best evaluated by the grade of activity and the stage of fibrosis 4. In the progression of MAFLD, the stage of liver fibrosis plays a key role 5, 6. Stage of liver fibrosis is also a strong predictor for disease-specific mortality in MAFLD, rather than other histologic features, i.e., steatosis, hepatocellular ballooning or lobular inflammation 7-9. Currently, liver biopsy is the gold standard to stage liver fibrosis 10. However, due to the possible acute complications and sampling errors of liver biopsy, this invasive diagnostic method is not the optimal choice in routine clinical practice 11.

Positron emission tomography (PET) is a molecular imaging modality, which has shown an important role in diagnosis, staging, assessment of response to treatment, and detecting recurrence of various types of cancers. ^18^F-fluorodeoxyglucose (^18^F-FDG), which is the most commonly used radiotracers for PET examinations, allows a direct quantification of glucose metabolism *in vivo* 12. The overall metabolic activity can be expressed semi-quantitatively as standardized uptake value (SUV). Recent studies showed that impaired hepatic glucose metabolism is associated with advanced fibrosis or cirrhosis 13, 14. However, the mean SUV or maximum SUV used in those studies neglect the heterogeneous distribution of liver histology characteristics 11, 15.

Radiomics 16, which allows the extraction of numerous quantitative features from medical imaging, so then may possibly reflect histological characteristics. Although radiomics, based on magnetic resonance imaging (MRI) or computed tomography (CT), have already been applied for diagnosing and staging fibrosis in some chronic liver diseases 17, 18, to our knowledge, there is no study on the radiomics based on ^18^F-FDG PET in MAFLD. Although the routine ^18^F-FDG PET has shown a potential role in the diagnosis and evaluation of fibrosis/cirrhosis in MAFLD, radiomics may help to scrutinize imaging data deeply to improve the performance. Since non-invasive tests for prediction of liver fibrosis have a poor diagnostic performance for detecting low levels of fibrosis 19, 20, it is important to explore the diagnostic capabilities of other non-invasive tests to diagnose low levels of liver fibrosis. Thus, the aim of our pilot study was to evaluate the performance of radiomics based on ^18^F-FDG PET in predicting liver fibrosis in individuals with MAFLD.

## Materials and Methods

### Study population and design

Subjects with biopsy-proven MAFLD, who underwent ^18^F-FDG PET/CT, were consecutively enrolled in this study. MAFLD was diagnosed according to newly proposed diagnostic criteria, namely evidence of fatty liver (on liver histology) in addition to one of following three criteria: 1) overweight or obesity, 2) type 2 diabetes mellitus (T2DM), or 3) presence of metabolic dysregulation 3. Individuals with a prior history of chronic hepatitis B or C, excessive alcohol consumption, or other chronic liver diseases were excluded from the study. The study protocol was approved by the local ethics committees. Written informed consent was obtained from each subject.

### Clinical and biochemical data

Clinical and biochemical data were obtained from all participants on the same day of liver biopsies. Hypertension was diagnosed if the subject had systolic blood pressure ≥130 mmHg or diastolic blood pressure ≥85 mmHg and/or if she/he assumed anti-hypertensive drugs. T2DM was diagnosed if the subject had fasting glucose levels ≥7.0 mmol/L, or glycosylated hemoglobin (HbA1c) ≥6.5% (≥48 mmol/mol), or a history of self-reported diabetes, and/or if she/he used any glucose-lowering drugs. Biochemical parameters, including serum levels of liver enzymes (alanine aminotransferase (ALT), aspartate aminotransferase (AST), and γ-glutamyltranspeptidase), total bilirubin, albumin, insulin, glucose, HbA1c, triglycerides, total cholesterol, low-density lipoprotein cholesterol and high-density lipoprotein cholesterol, were measured for each subject in the morning after an overnight fast.

### Liver histology

Percutaneous liver biopsies were performed under the guidance of ultrasound and all liver histology specimens were examined by a single experienced histopathologist, who was blinded to all participant's details. According to the NASH-Clinical Research Network Scoring System 10, liver biopsy specimens were assessed for steatosis (grades 0 to 3), ballooning (grades 0 to 2) and lobular inflammation (grades 0 to 3). Then, they were used to calculate the NAFLD activity score (NAS) by the unweighted sum 10. Liver fibrosis was assessed (grades 0 to 4) according to the Brunt's histologic criteria 21.

### ^18^F-FDG PET/CT scans

All participants were required to fast for at least 6 hours and the levels of serum glucose were less than 110 mg/dL before the ^18^F-FDG PET/CT scan. PET/CT images were acquired by a hybrid PET/CT scanner (GEMINI TF 64, Philips). For attenuation correction, a low-dose plain CT scan was performed from the skull base to the middle of thigh, with the following parameters: tube voltage=120 kV, tube current=249 mA, detector collimation=64 × 0.625 mm, pitch=0.829, tube rotation time=0.5 sec, slice thickness=5.0 mm. A three-dimension mode PET scan was performed approximately 1 hour later, after intravenous injection of ^18^F-FDG with a dose of 3.7 MBq/kg. The PET scan parameters were as follows: field of view (FOV) of 576 mm, matrix of 144 × 144, slice thickness and interval of 5.0 mm. The PET images were reconstructed using the ordered subset expectation maximization (OSEM) method (33 subsets per iteration). All collected data were transferred into Philips Extend Brilliance Workstation 3.0 to reconstruct PET, CT, and PET/CT fusion images, respectively.

### Radiomics features: extraction and selection

The whole process of radiomics features extraction was performed by the LIFEx version 6.30 software (http://www.lifexsoft.org) based on standardized practices 22. The PET images were imported into the software in the DICOM format. A whole liver region of interest (ROI) was manually drawn at the level of porta hepatis for each subject, by two radiologists in consensus (ZWC and YFZ, both with more than 7 years of experience), who were blinded to the clinical data. If there was any distinguishable abnormal lesion on the plain CT or PET images that would affect the results of radiomics analysis, the ROI slice of PET was moved up or down until there was no distinguishable abnormal lesion. Then, the images were handled by spatial resampling (to a voxel size of 4×4×4mm), intensity discretization (with 64 of grey levels and 0.3125 of bins) and intensity rescaling (the absolute method with min bound of 0 and max bound of 20). Finally, the software program calculates and extracts 60 PET 2D radiomics features automatically, including conventional indices, discretized indices, first order features, grey-level zone length matrix (GLZLM), grey-level run length matrix (GLRLM), neighborhood grey-level difference matrix (NGLDM) and grey-level co-occurrence matrix (GLCM) (**Table S1**). Another radiologist (KT, with more than 15 years of experience), who was blinded to the results of the first two radiologists, repeated all ROI identifications and feature extractions, as described above, for the assessment of inter-observer agreement.

Before feature selection, all of the extracted radiomics features were z-score standardized. The event per variable (EPV), defined by the ratio of the number of observations in the smaller of the two outcome groups relative to the number of variables, is a key factor for obtaining robust performance of prediction models. Lower EPV values in prediction model development are associated with poorer predictive performance 23, 24. According to an acceptable event per variable (EPV) value of 5 reported by previous studies 25, the method of max-relevance and min-redundancy (mRMR) 26 was used to select a subset of two features with mostly related to the output classes and low redundancy between them for further logistic regression. In addition, we also developed Support Vector Machine (SVM), Naive Bayes, 5-Nearest Neighbor and linear discriminant analysis models to classify each study participant based on the two selected features, respectively.

### Statistical analysis

All the statistical tests were performed in SPSS version 22 (IBM Corp.) and R version 3.6.3 (http://www.r-project.org/). Continuous variables were expressed as means ± standard deviation (SD) or medians with inter-quartile ranges. Categorical variables were expressed as numbers (and percentages). For all models, the prediction probability ≥0.5 was as a positive prediction outcome, otherwise was negative. The receiver operator characteristic (ROC) curve analysis was used for the binary classification and the area under ROC curve (AUROC), accuracy, sensitivity, specificity, as well as positive predictive value (PPV), negative predictive value (NPV), positive likelihood ratio (PLR), negative likelihood ratio (NLR) and diagnostic odds ratio were calculated to assess the predictive performance. The logistic regression model calibration was assessed by the Hosmer-Lemeshow goodness of fit test. Kappa statistics were generated to assess the inter-observer agreement. A two-sided p-value less than 0.05 was considered to be statistically significant for all statistical tests.

## Results

### Patients' characteristics

**Table 1** summarizes the baseline characteristics of individuals with biopsy-proven MAFLD, stratified by presence or absence of any histological stage of liver fibrosis. The mean (SD) age of the subjects was 38.5 (10.4) years and 17 of them were men. All the time-intervals between liver biopsy and PET/CT were less than 3 months. Among the 12 participants with liver fibrosis on liver biopsy, 10 subjects had F1 fibrosis and 2 subjects had F2 fibrosis. None of them had F3 or F4 fibrosis. As shown in the table, no significant differences were found in age, sex, metabolic comorbidities, laboratory parameters, as well as liver histology features (steatosis grade, ballooning grade, and lobular inflammation grade) between MAFLD subjects with and without fibrosis.

### Development of radiomics models

**Figure 1:**
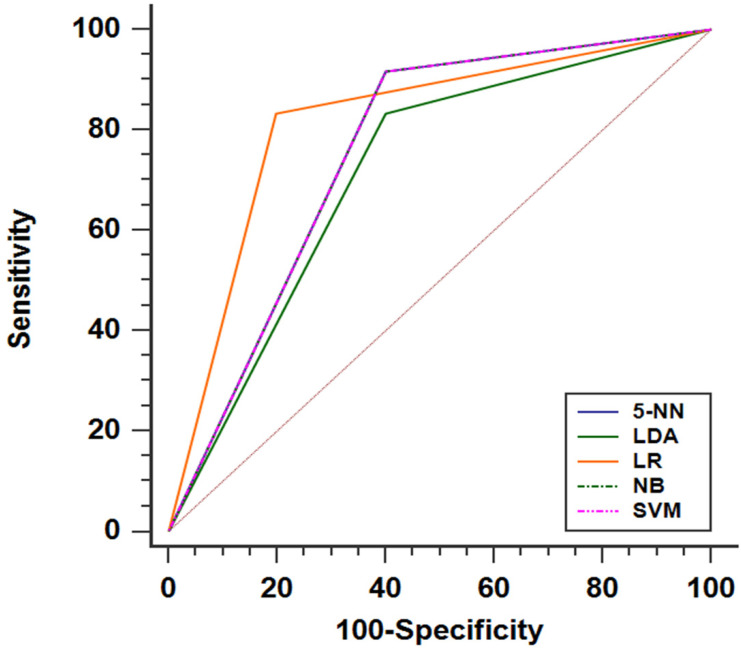
The performances of radiomics based on ^18^F-FDG PET models for predicting liver fibrosis (of any stage) presented as ROC curve. Note: The SVM, NB and 5-NN models had the same ROC performance. Abbreviations: LR: logistic regression; SVM: Support Vector Machine; NB: Naive Bayes; 5-NN: 5-Nearest Neighbor; LDA: linear discriminant analysis.

**Figure 2:**
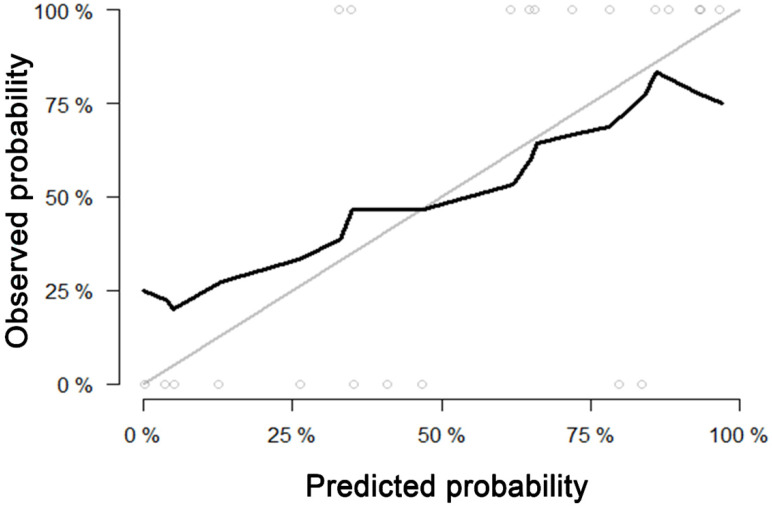
Calibration curve of the model built by logistic regression for predicting fibrosis. Calibration curve depict the calibration of model in terms of the agreement between the predicted probability and observed probability. The black solid line represents the performance of the model, which was closer to the diagonal grey solid line represents a better prediction. The Hosmer-Lemeshow test yielded a non-significant statistics for the model (p= 0.472), which suggested that there was no departure from perfect fit.

For predicting liver fibrosis in MAFLD, the coarseness of NGLDM and long-run emphasis (LRE) of GLRLM were selected for logistic regression to build the predictive model. The logistic regression model showed a good dichotomous prediction for fibrosis of any stage with an AUROC of 0.817 [95% confidence interval (CI), 0.595-0.947], sensitivity of 83.3%, specificity of 80%, and accuracy of 81.8%, respectively (**Table 2**). The ROC for predicting liver fibrosis is shown in **Fig. 1**. The Hosmer-Lemeshow goodness of fit showed a good calibration (p=0.472) (**Fig. 2**). The inter-observer agreement was excellent (Kappa coefficient=0.908).

The SVM, Naive Bayes and 5-Nearest Neighbor models yielded the same performance, showing a slightly worse prediction for fibrosis than the logistic regression model with an AUROC of 0.758 [95% CI, 0.531-0.913], sensitivity of 91.7%, specificity of 60.0%, and accuracy of 77.3%. The linear discriminant analysis model performed worst compared with the others. The linear discriminant analysis model AUROC, sensitivity, specificity and accuracy were 0.717 (95%CI, 0.487-0.886), 83.3%, 60% and 72.7%, respectively (**Fig. 1** and **Table 2**). Comparing the results obtained from the two sets of ROIs, the 5-Nearest Neighbor and linear discriminant analysis models showed perfect inter-observer agreements (both Kappa coefficients=0.999), and the SVM and Naive Bayes models also showed excellent inter-observer agreements (both Kappa coefficients=0.908).

## Discussion

In this pilot study, we found that radiomics based on ^18^F-FDG PET can be used with good diagnostic performance to diagnose the presence of early liver fibrosis in MAFLD. To our knowledge, this is the first study to use radiomics based on ^18^F-FDG PET to diagnose liver fibrosis in people with histologically proven MAFLD.

In contrast to other imaging techniques (CT, MRI and ultrasonography), PET can evaluate physiological and biochemical changes in cell metabolism in living tissues or organs in physiological states and diseases; noninvasively, dynamically and quantitatively at a molecular level. Previous small studies have shown the usefulness of ^18^F-FDG PET in some chronic liver diseases. In particular, the mean SUV was found to be significantly lower in patients with cirrhosis than in healthy controls 27, and collagen fiber deposition reduced the intrahepatic blood flow 28, thus leading to a decrease in ^18^F-FDG uptake. Experimentally, Pan et al. 29 combined ^18^F-FDG PET and gadolinium-ethoxybenzyl-diethylenetriamine-pentaacetic acid (Gd-EOB-DTPA) enhanced MRI to stage liver fibrosis in animal models by intraperitoneal injection of CCl_4_. These investigators found that the mean SUV value of ^18^F-FDG alone identified severe fibrosis, but did not distinguish between mild fibrosis and no fibrosis. Combining ^18^F-FDG PET and Gd-EOB-DTPA enhanced MRI had good accuracy for differentiating between fibrosis and no fibrosis, which was slightly lower than the performance of the model developed in this study (AUC ~0.80 vs. 0.82) 29. Unfortunately, due to the limited sample size of our pilot study, we did not develop a model to differentiate different fibrosis stages further as most of our MAFLD patients had F1 fibrosis.

MAFLD shows some heterogeneity 11. Mean SUV and maximum SUV are the most commonly used parameters with PET. However, mean SUV is influenced by hepatic fat content and the maximum SUV does not take into account the heterogeneity of the disease. Keramida et al. 30 suggested that the coefficient of variation of the regional ratio of maximum SUV to mean SUV may be a marker of hepatic fat distribution heterogeneity. Besides, it has also been established that radiomics is a powerful tool for the assessment of disease heterogeneity 31. In our study, the coarseness of NGLDM and LRE of GLRLM were selected to build the best predictive model. All of the features can reflect the distribution or adjacent relationships of pixels in the images. By describing the heterogeneity of pixels on the image, we may be able to understand the heterogeneity of liver histological characteristics but further work in this area is needed.

In this pilot study, whole liver ROIs were manually drawn by hand for analysis. Although manual delineation may be subjective, the liver is well circumscribed, which limits subjective differences between individuals and it is noteworthy that there was an excellent inter-observer agreement in our study. For a fixed size ROI, the size of the ROI will affect the extracted features, and the optimal size is not known. Thus, the size and placement of fixed ROIs may introduce more subjectivity and selection bias, due to the heterogeneity of liver histological characteristics in MAFLD. Nevertheless, it would have been preferable to have an accurate automatic segmentation method rather than the manual method used in our pilot study.

The small sample size of the study is the most important limitation of our pilot study, which may introduce bias and limits power. However, to our knowledge, this study is the first study to date to assess the performance of ^18^FDG PET-based radiomics for predicting liver fibrosis in individuals with MAFLD. In contrast to some previous studies that used ultrasonography or other imaging techniques for diagnosing MAFLD 32, 33, the most important strength of our study is that the diagnosis and staging of MAFLD was based on liver biopsy and we have identified a substantial number of subjects with early liver fibrosis (stage F1). Our results suggest the feasibility of PET for predicting early liver fibrosis in MAFLD but further studies are needed in this research field. Due to the small sample size of the study, no validation was conducted. However, because of the influence of EPV, we limited the number of selected features to make our predictive model as stable as possible, although that needs to be verified in further studies. We chose the mRMR method in order to control the number of selected features rather than other selection methods, e.g. the least absolute shrinkage and selection operator. Although the developed model in our pilot study may not be optimal, the results suggest that radiomics-based ^18^F-FDG PET may be a promising tool for the assessment of early liver fibrosis in MAFLD.

In conclusion, the results of this pilot study suggest that ^18^F-FDG PET radiomics may offer a potential tool for the assessment of early liver fibrosis in individuals with biopsy-proven MAFLD, which is worthy to be further evaluation in larger multicenter studies in different ethnic groups.

## Supplementary Material

Supplementary table.

## Figures and Tables

**Table 1:** Baseline characteristics of individuals with MAFLD stratified by the presence of any histological stage of fibrosis

	With fibrosis (n=12)	Without fibrosis (n=10)	*p*-value
***Demographics***			
Age, years	37.7 ± 9.4	38.8 ± 11.4	0.794
Male sex, n (%)	11 (91.7%)	6 (60.0%)	0.135
**Metabolic comorbidities**			
Type 2 diabetes, n (%)	1 (8.3%)	2 (20.0%)	0.571
Hypertension, n (%)	2 (16.7%)	1 (10.0%)	0.999
BMI, kg/m^2^	27.1 ± 3.3	27.8 ± 2.4	0.576
**Laboratory parameters**			
Alanine aminotransferase, IU/L	127.2 ± 71.4	88.1 ± 52.6	0.167
Aspartate aminotransferase, IU/L	60.7 ± 26.8	48.8 ± 20.9	0.267
γ-glutamyltranspeptidase, IU/L	89.5 ± 50.6	59.6 ± 38.3	0.140
Albumin, g/L	48.2 ± 3.7	48.2 ± 4.0	0.994
Total bilirubin, μmol/L	13.7 ± 3.23	12.2 ± 3.0	0.290
Fasting glucose, mmol/L	5.1 (4.8-5.9)	5.3 (5.1-5.6)	0.628
Fasting insulin, pmol/L	112.4 (71.7-211.1)	96.8 (78.4-325.5)	0.923
Glycosylated hemoglobin, mmol/mol	36 (33-46)	37 (32-42)	0.539
Total cholesterol, mmol/L	5.0 ± 1.5	5.6 ± 1.6	0.384
Triglycerides, mmol/L	2.0 ± 1.1	2.1 ± 1.1	0.868
HDL-cholesterol, mmol/L	1.0 ± 0.2	1.1 ± 0.2	0.059
LDL-cholesterol, mmol/L	3.2 ± 1.1	3.5 ± 1.2	0.567
***Liver histology features***			
**Steatosis grade, n (%)**			0.091
S1	2 (16.7%)	4 (40.0%)	
S2	8 (66.7%)	2 (20.0%)	
S3	2 (16.7%)	4 (40.0%)	
**Ballooning grade, n (%)**			0.065
B0	0 (0.0%)	3 (30.0%)	
B1	7 (58.3%)	6 (60.0%)	
B2	5 (41.7%)	1 (10.0%)	
**Lobular inflammation grade, n (%)**			0.107
L0	0 (0.0%)	3 (30.0%)	
L1	9 (75.0%)	4 (40.0%)	
L2	3 (25.0%)	3 (30.0%)	
L3	0 (0.0%)	0 (0.0%)	

Note: Continuous variables were expressed as means ± standard deviation or medians with interquartile ranges. Categorical variables were expressed as number (percentages).

**Table 2:** Performance of ^18^F-FDG PET radiomics for diagnosing any stage of liver fibrosis, in different models, in biopsy-proven MAFLD

	LR	SVM	NB	5-NN	LDA
AUROC (95%CI)	0.817 (0.595-0.947)	0.758 (0.531-0.913)	0.758 (0.531-0.913)	0.758 (0.531-0.913)	0.717 (0.487-0.886)
Sensitivity, % (n/N)	83.3 (10/12)	91.7 (11/12)	91.7 (11/12)	91.7 (11/12)	83.3 (10/12)
Specificity, % (n/N)	80.0 (8/10)	60.0 (6/10)	60. 0 (6/10)	60.0 (6/10)	60.0 (6/10)
Accuracy, % (n/N)	81.8 (18/22)	77.3 (17/22)	77.3 (17/22)	77.3 (17/22)	72.7 (16/22)
PPV, % (n/N)	83.3 (10/12)	73.3 (11/15)	73.3 (11/15)	73.3 (11/15)	71.4 (10/14)
NPV, % (n/N)	80.0 (8/10)	85.7 (6/7)	85.7 (6/7)	85.7 (6/7)	75.0 (6/8)
PLR	4.17	2.29	2.29	2.29	2.08
NLR	0.21	0.14	0.14	0.14	0.28
Diagnostic odds ratio	19.9	16.4	16.4	16.4	7.4

Abbreviations: ^18^F-FDG, fluorine-18-fluorodeoxyglucose; PET, positron emission tomography; MAFLD, metabolic dysfunction-associated fatty liver disease; LR: logistic regression; SVM: Support Vector Machine; NB: Naive Bayes; 5-NN: 5-Nearest Neighbor; LDA: linear discriminant analysis; AUROC, area under the receiver operating characteristic curve; CI, confidence interval; PPV, positive predictive value; NPV, negative predict value; PLR, positive likelihood ratio; NLR, negative likelihood ratio.

## Abbreviations

ALT: alanine aminotransferase
AST: aspartate aminotransferase
AUROC: area under ROC curve
CT: computed tomography
CI: confidence interval
EPV: event per variable
^18^F-FDG: ^18^F-fluorodeoxyglucose
FOV: field of view
GLCM: grey-level co-occurrence matrix
GLRLM: grey-level run length matrix
GLZLM: grey-level zone length matrix
MAFLD: metabolic dysfunction-associated fatty liver disease
MRI: magnetic resonance imaging
mRMR: max-relevance and min-redundancy
NAFLD: non-alcoholic fatty liver disease
NAS: NAFLD activity score
NGLDM: neighborhood grey-level difference matrix
OSEM: ordered subset expectation maximization
PET: positron emission tomography
ROC: receiver operator characteristic
ROI: region of interest
SUV: standardized uptake value
SVM: Support Vector Machine
T2DM: type 2 diabetes mellitus
