# Supplementary material for: Radiomics based on fluoro-deoxyglucose positron emission tomography predicts liver fibrosis in biopsy-proven MAFLD: a pilot study
Source: Int J Med Sci. 2021 Sep 7;18(16):3624–30. doi: 10.7150/ijms.64458 (PMC8579290; doi:10.7150/ijms.64458)
Supplement: Supplementary file 1 — Supplementary table. [file ijmsv18p3624s1.pdf]

Table S1. The extracted radiomics features

Conventional (n=11)	SUVbwmin<br>SUVbwmean<br>SUVbwstd<br>SUVbwmax<br>SUVbwQ1<br>SUVbwQ2<br>SUVbwQ3<br>SUVbwSkewness<br>SUVbwKurtosis<br>SUVbwExcessKurtosis<br>TLG
Discretized (n=11)	SUVbwmin<br>SUVbwmean<br>SUVbwstd<br>SUVbwmax<br>SUVbwQ1<br>SUVbwQ2<br>SUVbwQ3<br>SUVbwSkewness<br>SUVbwKurtosis<br>SUVbwExcessKurtosis<br>TLG
Histogram (n=6)	Skewness<br>Kurtosis<br>ExcessKurtosis<br>Entropy_log10<br>Entropy_log2<br>Energy
GLCM (n=7)	Homogeneity<br>Energy<br>Contrast<br>Correlation<br>Entropy_log10<br>Entropy_log2<br>Dissimilarity
GLRLM (n=11)	SRE<br>LRE<br>LGRE<br>HGRE<br>SRLGE<br>SRHGE<br>LRLGE<br>LRHGE

	GLNU<br>RLNU<br>RP
NGLDM (n=3)	Coarseness<br>Contrast<br>Busyness
GLZLM (n=11)	SZE<br>LZE<br>LGZE<br>HGZE<br>SZLGE<br>SZHGE<br>LZLGE<br>LZHGE<br>GLNU<br>ZLNU<br>ZP

Detailed explanations and equations for the radiomic features were listed in the website: https://www.lifexsoft.org/index.php/resources/19-texture/radiomic-features?filter_tag[0]=

Abbreviations：SUV: standardized uptake value; TLG: total lesion glycolysis; GLCM: grey-level co-occurrence matrix; GLRLM: grey-level run length matrix; NGLDM: neighborhood grey-level difference matrix; GLZLM: grey-level zone length matrix; SRE: short-run emphasis; LRE: long-run emphasis; LGRE: low gray-level run emphasis; HGRE: high gray-level run emphasis; SRLGE: short-run low gray-level emphasis; SRHGE: short-run high gray-level emphasis; LRLGE: long-run low gray-level emphasis; LRHGE: long-run high gray-level emphasis; GLNU: gray-level non-uniformity; RLNU: run length non-uniformity; RP: run percentage; SZE: short-zone emphasis; LZE: Long-Zone Emphasis; LGZE: low gray-level zone emphasis; HGZE: high gray-level zone emphasis; SZLGE: short-zone low gray-level emphasis; SZHGE: short-zone high gray-level emphasis; LZLGE: long-zone low gray-level emphasis; LZHGE: long-zone high gray-level emphasis; GLNU: gray-level non-uniformity; ZLNU: zone length non-uniformity; ZP: zone percentage.
